# Site-specific prognosis and temporal trends in extranodal marginal zone lymphoma patients in the United States

**DOI:** 10.1038/s41408-025-01424-4

**Published:** 2025-11-21

**Authors:** Malak Munir, John L. Vaughn, Sravani Rimmalapudi, Narendranath Epperla

**Affiliations:** 1 Karsh Division of Gastroenterology and Hepatology, Cedars-Sinai Medical Center, Los Angeles, CA USA; 2 Division of Hematology & Oncology, NYU Grossman Long Island School of Medicine, New York, NY USA; 3 Division of Hematology and Hematologic Malignancies, Huntsman Cancer Institute, University of Utah, Salt Lake City, UT USA

**Keywords:** Health care, Medical research

**To the Editor**,

Marginal zone lymphoma (MZL) is an indolent B-cell non-Hodgkin lymphoma that accounts for ~7% of all non-Hodgkin lymphomas [1]. MZL is a heterogeneous disease and includes three subtypes, nodal MZL, splenic MZL, and extranodal MZL of mucosa-associated lymphoid tissue (EMZL). The management of EMZL has evolved over time, with the introduction of rituximab, bendamustine, Bruton Tyrosine Kinase Inhibitors (BTKi), and lenalidomide [2–5]. Clinical presentation varies by anatomic site, but prognostic significance has not been well-characterized [6]. We conducted a population-based cohort study using data from the Surveillance, Epidemiology, and End Results-17 (SEER) database to examine survival outcomes by anatomic site and era of diagnosis in patients with EMZL over a 20-year period from 2001–2021 in USA.

We included adults aged 18 years and older with pathologically confirmed EMZL defined by ICD-O-3 codes (9699/3), diagnosed between 2001 and 2021. We excluded patients with central nervous system involvement and those with primary nodal disease, as well as patients whose site of diagnosis was spleen (Supplementary Table S4). Patients were stratified into three cohorts based on diagnosis period to reflect 3 treatment eras 2001–2008 (rituximab/pre-bendamustine era), 2009–2016 (to reflect bendamustine era), and 2017–2021 (to reflect novel therapies including BTKi and lenalidomide). Data were obtained from the SEER-17 database, representing ~26.5% of the US cancer patient population. We used SEER’s person-level record linkage to identify patients with histologic transformation (HT) to DLBCL (9680/3).

The primary outcomes were relative survival (RS), overall survival (OS), and EMZL-specific death. RS was defined as the ratio of the proportion of observed survivors in a cohort of cancer patients to the proportion of expected survivors in a comparable set of cancer-free individuals for the period 2001–2020. OS was defined as time from diagnosis to death from any cause, EMZL-specific death was defined as death from lymphoma, with death from other causes treated as a competing event. Covariates included age, sex, race/ethnicity (Non-Hispanic White, Non-Hispanic Black, Hispanic), anatomic site (gastric, salivary gland, bone/connective tissue, ocular, oropharyngeal, other gastrointestinal, skin, genitourinary, thyroid, pulmonary, breast, unknown, other), AJCC stage, and period of diagnosis. Baseline characteristics were compared using Kruskal-Wallis and chi-square tests. Adjusted Cox models estimated hazard ratios for OS. Adjusted Fine-Gray competing risks regression estimated subdistribution hazard ratios for EMZL-specific death, with death from other causes as a competing event. 3-year RS was estimated using the Pohar-Perme method using SEER*Stat, analyses were conducted in R version 4.2.2, *P* < 0.05 was considered significant.

The study was conducted in compliance with the Declaration of Helsinki. Given the nature of the study (population based study from a public repository), this study was IRB exempt. As this was a retrospective study, informed consent was waived.

We included 15,499 patients with EMZL, of whom 5252 were diagnosed in 2001–2008, 6284 in 2009–2016, and 3963 in 2017–2021. The median age was 64 (range, 54 - 74) years  with 55.6% females, 65.2% NHW, and 8.1% NHB, and median follow-up duration of 6.5 years. The most common anatomic sites were Gastric (32.5%), Ocular (13.2%), and Skin (12%). 48.3% of patients had stage I disease. Among the 4534 patients who died during follow-up, 901 (19.9%) died from EMZL-specific causes and 3633 (80.1%) died from other causes. The proportion of EMZL-specific deaths increased with stage: Stage I 14.9%, Stage II 27.8%, Stage III 37.6%, and Stage IV 32.9%. A summary of patient baseline characteristics is reported in Table 1.

**Table 1:** Baseline characteristics of patients diagnosed with EMZL in the United States 2001–2021.

Characteristic	Overall (*N* = 15,499)	2001–2008 (*n* = 5252)	2009–2016 (*n* = 6284)	2017–2021 (*n* = 3963)	*P*-value
Median Follow-up, years (range)	6.5 (2.9, 11.4)	13.5 (6.8, 16.4)	7.3 (5.5, 9.8)	2.2 (0.9, 3.5)	<0.001
Median age in years (range)	64 (54, 74)	65 (53, 75)	64 (54, 73)	65 (55, 74)	0.017
Sex					0.4
Female	8618 (56%)	2960 (56%)	3473 (55%)	2185 (55%)	
Male	6881 (44%)	2292 (44%)	2811 (45%)	1778 (45%)	
Race/Ethnicity					<0.001
Hispanic	4150 (27%)	1210 (23%)	1767 (28%)	1173 (30%)	
NHB	1248 (8.1%)	375 (7.1%)	531 (8.5%)	342 (8.6%)	
NHW	10,101 (65%)	3667 (70%)	3986 (63%)	2448 (62%)	
Anatomic site					<0.001
Bone/Connective Tissue	382 (2.5%)	119 (2.3%)	153 (2.4%)	110 (2.8%)	
Breast	576 (3.7%)	177 (3.4%)	231 (3.7%)	168 (4.2%)	
Gastric	5037 (32%)	1899 (36%)	2007 (32%)	1131 (29%)	
Genitourinary	265 (1.7%)	78 (1.5%)	101 (1.6%)	86 (2.2%)	
Ocular	2048 (13%)	724 (14%)	796 (13%)	528 (13%)	
Oropharyngeal	618 (4.0%)	219 (4.2%)	253 (4.0%)	146 (3.7%)	
Other	141 (0.9%)	26 (0.5%)	63 (1.0%)	52 (1.3%)	
Other GI	1485 (9.6%)	501 (9.5%)	591 (9.4%)	393 (9.9%)	
Pulmonary	1498 (9.7%)	455 (8.7%)	590 (9.4%)	453 (11%)	
Salivary Gland	1182 (7.6%)	455 (8.7%)	485 (7.7%)	242 (6.1%)	
Skin	1855 (12%)	468 (8.9%)	829 (13%)	558 (14%)	
Thyroid	293 (1.9%)	116 (2.2%)	122 (1.9%)	55 (1.4%)	
Unknown	119 (0.8%)	15 (0.3%)	63 (1.0%)	41 (1.0%)	
Stage					<0.001
Stage I	7481 (48%)	2302 (44%)	3667 (58%)	1512 (38%)	
Stage II	1238 (8.0%)	352 (6.7%)	598 (9.5%)	288 (7.3%)	
Stage III	216 (1.4%)	72 (1.4%)	118 (1.9%)	26 (0.7%)	
Stage IV	1196 (7.7%)	349 (6.6%)	532 (8.5%)	315 (7.9%)	
Unknown	5368 (35%)	2177 (41%)	1369 (22%)	1822 (46%)	
Radiation therapy					<0.001
None/Unknown	10,140 (65%)	3618 (69%)	4063 (65%)	2459 (62%)	
Radiation	5359 (35%)	1634 (31%)	2221 (35%)	1504 (38%)	
Chemotherapy					<0.001
No/Unknown	12,748 (82%)	4033 (77%)	5125 (82%)	3590 (91%)	
Yes	2751 (18%)	1219 (23%)	1159 (18%)	373 (9.4%)	
Transformation to DLBCL	256 (1.7%)	110 (2.1%)	118 (1.9%)	28 (0.7%)	<0.001
Outcome					<0.001
Alive	10,965 (71%)	2608 (50%)	4701 (75%)	3656 (92%)	
EMZL-specific death	901 (5.8%)	513 (9.8%)	309 (4.9%)	79 (2.0%)	
Death from other causes	3633 (23%)	2131 (41%)	1274 (20%)	228 (5.8%)	

Continuous variables are presented as Median (Q1, Q3); Categorical variables are presented as *n* (%).

The 3-year age-standardized relative survival for patients with EMZL improved from 95.1% (95% CI: 94.4–95.7%) in 2001–2008 to 96.2% (95.6–96.8%) in 2009–2016 to 96.7% (95.7–97.5%) in 2017–2021. In competing risk analysis, the 3-year cumulative incidence of EMZL-specific death decreased progressively across therapeutic eras: 3.7% in 2001–2008, 2.9% in 2009–2016, and 2.5% in 2017–2021 (*P* < 0.001) (Fig. 1A). The cumulative incidence of death from other causes remained similar across periods: 7.8%, 6.8%, and 7.3%, respectively (*P* = 0.125).

**Fig. 1:**
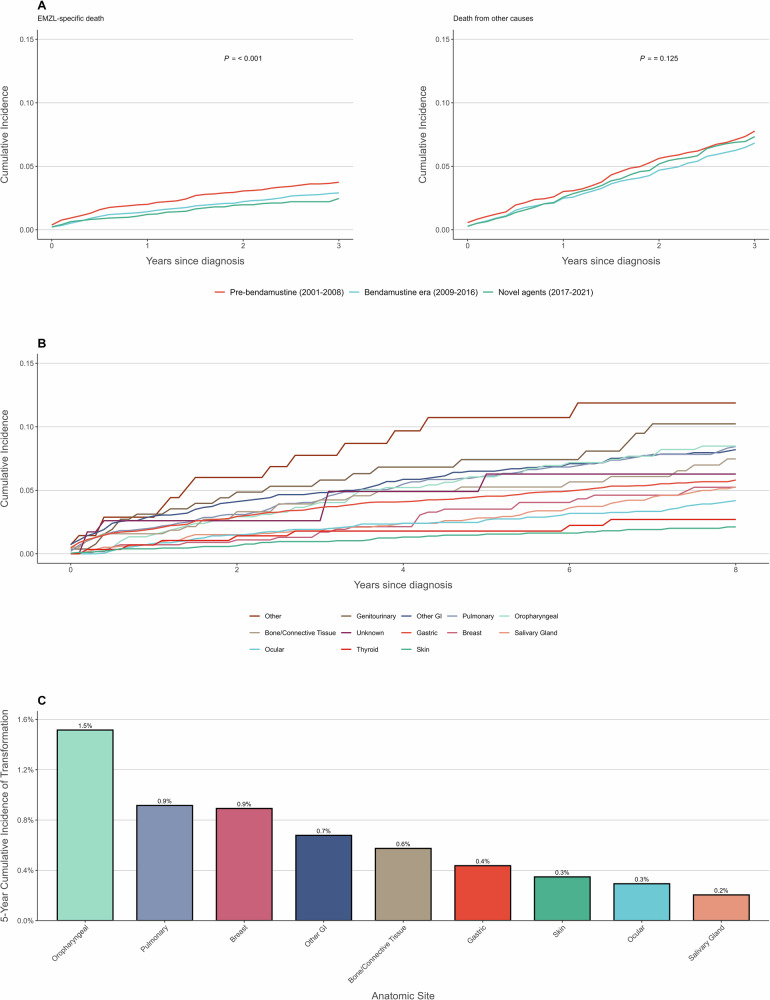
Survival outcomes and transformation risk in extranodal marginal zone lymphoma. **A** Cumulative incidence of EMZL-specific death (left) and death from other causes (right) by therapeutic era. **B** Cumulative incidence of EMZL-specific death by anatomic site. **C** Five-year cumulative incidence of transformation to diffuse large B-cell lymphoma by anatomic site.

After adjusting for age, sex, race/ethnicity, anatomic site, and stage, each additional year of diagnosis was associated with improved OS (HR 0.98 per year, 95% CI: 0.98–0.99, *P* < 0.001) (Supplementary Table S1) and reduced EMZL-specific mortality (SHR 0.94 per year, 95% CI: 0.93–0.95, *P* < 0.001), corresponding to relative risk reductions of 28% and 72%, respectively, over the 20-year study period.

The cumulative incidence functions of EMZL-specific death varied significantly by anatomic site (*P* < 0.001). The highest 5-year cumulative incidence was observed in patients in the Other category (10.7%), Genitourinary (7.4%), Other GI (6.5%), and Pulmonary (6.1%), while the lowest was in Skin (1.5%) (Supplementary Table S2, Fig. 1B).

In multivariable analysis, compared to gastric EMZL, patients with elevated risk included Other GI (SHR 1.47, 95% CI: 1.19–1.83, *P* < 0.001), Pulmonary (SHR 1.46, 95% CI: 1.17–1.82, *P* < 0.001). Patients with lower risk included Skin (SHR 0.55, 95% CI: 0.38–0.78, *P* < 0.001). Borderline statistically significant associations were observed for Bone/Connective Tissue (SHR 1.43, 95% CI: 0.97–2.1, *P* = 0.07), Oropharyngeal (SHR 1.33, 95% CI: 0.97–1.82, *P* = 0.08), Genitourinary (SHR 1.47, 95% CI: 0.96–2.25, *P* = 0.07) (Supplementary Table S3).

We identified 256 patients (1.65%) who experienced histologically confirmed transformation to diffuse large B-cell lymphoma, with a median time to transformation of 4 years. Among the 4534 patients who died during follow-up, 125 (2.8%) had HT prior to death. Among patients who died of EMZL-specific causes, the proportion with HT was higher (79/901, 8.8%) compared to those who died of other causes (46/3633, 1.3%).

Transformation risk varied significantly by anatomic site (Gray’s test *P* < 0.001), with the highest 5-year cumulative incidence observed in oropharyngeal (1.5%), pulmonary (0.9%), and breast (0.9%), while the lowest rates were in salivary gland (0.2%), ocular (0.3%), and cutaneous sites (0.3%) (Fig. 1C). In multivariable competing risk analysis oropharyngeal (SHR 3.74, 95% CI: 2.33–6.01), bone/connective tissue (SHR 3.33, 95% CI: 1.81–6.12), and genitourinary sites (SHR 3.25, 95% CI: 1.61–6.58) showed significantly elevated HT risk compared to gastric EMZL.

In this population-based analysis of patients with EMZL, we found significant survival improvements over time and differences in prognosis by anatomic site, consistent with recent population-based studies reporting improving trends in OS for EMZL compared to splenic and nodal marginal zone lymphoma subtypes [7]. The 3 year cumulative incidence of EMZL-specific mortality decreased from 3.7% in 2001–2008 to 2.5% in 2017–2021 with a relative risk reduction of 72% over the course of 20 years, consistent with the indolent course of EMZL [8], likely reflecting the broader implementation of rituximab-based therapies and novel agents (BTK inhibitors/lenalidomide) [2, 3, 9]. Histologic transformation to DLBCL occurred in 1.65% of patients, with significant variation by anatomic site; oropharyngeal, bone/connective tissue, and genitourinary sites demonstrated 3-4 fold higher transformation risk compared to gastric EMZL, in-line with the reported rates in the literature [8, 10].

The marked variability in outcomes by primary site supports the concept that EMZL represents a collection of distinct clinicopathologic entities rather than a single disease [11]. Current treatment approaches are predominantly derived from gastric EMZL studies, with extrapolation to non-gastric sites often based on limited evidence, despite clear differences in treatment response rates ranging from 27% for some therapies to >90% for others depending on anatomic location [12]. This approach fails to account for the prognostic differences across anatomic sites. Previous population-based studies consistently document 5-year OS rates exceeding 90% for gastric EMZL, due to both its association with *H. pylori* infection (where eradication therapy achieves 70–80% complete remission rates) and recognition facilitating earlier diagnosis [13].

In our analysis, non-gastric gastrointestinal and pulmonary EMZL had significantly higher EMZL mortality risk compared to gastric EMZL, consistent with previous studies [6, 7]. These sites lack effective, site-specific, first-line therapy equivalent to *H. pylori* eradication and display more aggressive behavior, with increased likelihood of advanced-stage disease or multifocal involvement [7]. Notably, cutaneous EMZL had significantly better outcomes than gastric disease, consistent with the other studies, reflecting its excellent response to local therapy [7, 14].

Limitations of our study are mainly inherent to the SEER database which lacks detailed treatment information. Additionally, important biological factors such as *H. pylori* status, autoimmune associations, and molecular characteristics that might influence prognosis are not captured. Despite these limitations, the findings of this study provide compelling evidence for risk stratification based on anatomic site and highlights the need for site-specific approaches to this heterogeneous disease.

Our analysis highlights significant improvements in EMZL survival over the past two decades and identify substantial prognostic heterogeneity by anatomic site and supports the need for site-specific approaches to EMZL management. Current prognostic indices such as the MALT-IPI and Revised MALT-IPI [15], while validated for both gastric and non-gastric disease, are based primarily on patient factors rather than site-specific classification. There is a need for increased clinical awareness of EMZL at high-risk anatomic locations and inclusion of lymphoma in the differential diagnosis for patients with persistent symptoms at these sites.

## Supplementary information

Supplementary Appendix

## Data Availability

This study used publicly available data which can be accessed through the Surveillance, Epidemiology, and End Results (SEER)-17 database.
