# Supplementary material for: Site-specific prognosis and temporal trends in extranodal marginal zone lymphoma patients in the United States
Source: Blood Cancer J. 2025 Nov 21;15(1):201. doi: 10.1038/s41408-025-01424-4 (PMC12638744; doi:10.1038/s41408-025-01424-4)
Supplement: Supplementary file 1 — Supplementary Appendix [file 41408_2025_1424_MOESM1_ESM.docx]

**Supplemental Appendix**

**Table of Contents**

[Table S1. Cox Proportional Hazards Model of OS with Year of Diagnosis as a Continuous Variable 2](#_Toc210423797)

[Table S2. Cumulative Incidence of EMZL-Specific Death by Anatomic Site 3](#_Toc210423798)

[Table S3. SHR for EMZL-specific Death from Competing Risk Regression 4](#_Toc210423799)

[Table S4. Inclusion and Exclusion Criteria Based on ICD-10 Site Codes 6](#_Toc210423800)

# Table S1. Cox Proportional Hazards Model of Overall Survival with Year of Diagnosis as a Continuous Variable

| **Covariable** | **HR** | **95% CI** | **P value** |
| --- | --- | --- | --- |
| Year of Diagnosis (per year) | 0.98 | 0.98-0.99 | <0.001 |
| Age (per year) | 1.09 | 1.09-1.09 | <0.001 |
| **Sex (ref: Female)** |  |  |  |
| Male | 1.37 | 1.29-1.46 | <0.001 |
| **Race/Ethnicity (ref: Hispanic)** |  |  |  |
| Non-Hispanic Black | 1.56 | 1.38-1.77 | <0.001 |
| Non-Hispanic White | 1.13 | 1.05-1.22 | 0.001 |
| **Anatomic Site (ref: Gastric)** |  |  |  |
| Salivary Gland | 0.91 | 0.8-1.03 | 0.124 |
| Bone/Connective Tissue | 0.88 | 0.72-1.06 | 0.178 |
| Ocular | 0.79 | 0.72-0.88 | <0.001 |
| Oropharyngeal | 0.93 | 0.8-1.08 | 0.353 |
| Other GI | 0.99 | 0.89-1.1 | 0.862 |
| Skin | 0.65 | 0.57-0.74 | <0.001 |
| Genitourinary | 0.78 | 0.62-0.98 | 0.031 |
| Thyroid | 0.88 | 0.7-1.11 | 0.273 |
| Pulmonary | 1.12 | 1.01-1.24 | 0.028 |
| Breast | 0.95 | 0.81-1.12 | 0.568 |
| Unknown | 0.98 | 0.69-1.38 | 0.890 |
| Other | 1.12 | 0.84-1.49 | 0.431 |
| **Stage (ref: Stage I)** |  |  |  |
| Stage II | 1.31 | 1.17-1.46 | <0.001 |
| Stage III | 1.92 | 1.57-2.35 | <0.001 |
| Stage IV | 1.63 | 1.46-1.81 | <0.001 |
| Stage Unknown | 1.17 | 1.09-1.26 | <0.001 |

# Table S2. Cumulative Incidence of EMZL-Specific Death by Anatomic Site

| **Site** | **Total Cases** | **EMZL Deaths** | **5-Year CIF (%)** |
| --- | --- | --- | --- |
| Gastric | 5,037 | 294 | 4.5 |
| Ocular | 2,048 | 88 | 2.7 |
| Skin | 1,855 | 34 | 1.5 |
| Pulmonary | 1,498 | 124 | 6.1 |
| Other Gastrointestinal | 1,485 | 120 | 6.5 |
| Salivary Gland | 1,182 | 66 | 2.8 |
| Oropharyngeal | 618 | 50 | 6.1 |
| Breast | 576 | 38 | 3.5 |
| Bone/Connective Tissue | 382 | 30 | 5.3 |
| Thyroid | 293 | 11 | 1.8 |
| Genitourinary | 265 | 25 | 7.4 |
| Other | 141 | 14 | 10.7 |
| Unknown | 119 | 7 | 6.3 |

CIF = cumulative incidence function; EMZL = extranodal marginal zone lymphoma

# Table S3 Subdistribution Hazard Ratios for EMZL-specific Death from Competing Risk Regression

| **Covariable** | **SHR** | **95% CI** | ***P*-value** |
| --- | --- | --- | --- |
| Age (per year) | 1.06 | 1.05-1.06 | <0.001 |
| **Sex (ref: Female)** |  |  |  |
| Male | 1.24 | 1.08-1.42 | 0.003 |
| **Race/Ethnicity (ref: Hispanic)** |  |  |  |
| Non-Hispanic Black | 1.20 | 0.91-1.58 | 0.200 |
| Non-Hispanic White | 0.94 | 0.8-1.1 | 0.440 |
| **Anatomic Site (ref: Gastric)** |  |  |  |
| Breast | 1.28 | 0.9-1.8 | 0.170 |
| Genitourinary | 1.47 | 0.96-2.25 | 0.074 |
| Ocular | 0.86 | 0.67-1.09 | 0.210 |
| Oropharyngeal | 1.33 | 0.97-1.82 | 0.078 |
| Salivary Gland | 1.10 | 0.84-1.45 | 0.480 |
| Bone/Connective Tissue | 1.43 | 0.97-2.1 | 0.070 |
| Other | 1.58 | 0.9-2.78 | 0.110 |
| Other GI | 1.47 | 1.19-1.83 | <0.001 |
| Pulmonary | 1.46 | 1.17-1.82 | <0.001 |
| Thyroid | 0.81 | 0.45-1.48 | 0.500 |
| Skin | 0.55 | 0.38-0.78 | 0.001 |
| Unknown | 0.79 | 0.37-1.7 | 0.550 |
| **Stage (ref: Stage I)** |  |  |  |
| Stage Stage II | 2.10 | 1.66-2.65 | <0.001 |
| Stage Stage III | 4.13 | 2.86-5.96 | <0.001 |
| Stage Stage IV | 2.97 | 2.41-3.67 | <0.001 |
| Stage Unknown | 1.55 | 1.32-1.82 | <0.001 |
| **Period of Diagnosis (ref: Bendamustine 2009-2016)** |  |  |  |
| Novel agents (2017-2021) | 0.75 | 0.58-0.96 | 0.024 |
| Pre-bendamustine (2001-2008) | 1.55 | 1.34-1.78 | <0.001 |

SHR = subdistribution hazard ratio; CI = confidence interval

# Table S4. Inclusion and Exclusion Criteria Based on ICD-10 Site Codes

| **Category** | **Total Cases** | **ICD-10 Codes** |
| --- | --- | --- |
| **Excluded** | | |
| Lymph Nodes | 7,914 | C77.0-Lymph nodes of head, face & neck; C77.1-Intrathoracic lymph nodes; C77.2-Intra-abdominal lymph nodes; C77.3-Lymph nodes of axilla or arm; C77.4-Lymph nodes of inguinal region or leg; C77.5-Pelvic lymph nodes; C77.8-Lymph nodes of multiple regions; C77.9-Lymph node, NOS |
| Other | 1,271 | C42.0-Blood; C42.1-Bone marrow; C42.4-Hematopoietic system, NOS |
| Central Nervous System | 116 | C70.0-Cerebral meninges; C70.9-Meninges, NOS; C71.0-Cerebrum; C71.1-Frontal lobe; C71.2-Temporal lobe; C71.3-Parietal lobe; C71.4-Occipital lobe; C71.5-Ventricle, NOS; C71.6-Cerebellum, NOS; C71.8-Overlapping lesion of brain; C71.9-Brain, NOS; C72.0-Spinal cord; C72.3-Optic nerve; C72.8-Overlapping lesion of brain & CNS; C72.9-Nervous system, NOS |
| Spleen | 34 | C42.2-Spleen |
| **Included** | | |
| Gastric | 5,047 | C16.0-Cardia, NOS; C16.1-Fundus of stomach; C16.2-Body of stomach; C16.3-Gastric antrum; C16.4-Pylorus; C16.5-Lesser curvature of stomach NOS; C16.6-Greater curvature of stomach NOS; C16.8-Overlapping lesion of stomach; C16.9-Stomach, NOS |
| Ocular | 2,072 | C69.0-Conjunctiva; C69.1-Cornea, NOS; C69.2-Retina; C69.3-Choroid; C69.4-Ciliary body; C69.5-Lacrimal gland; C69.6-Orbit, NOS; C69.8-Overlapping lesion of eye and adnexa; C69.9-Eye, NOS |
| Skin | 1,890 | C44.0-Skin of lip, NOS; C44.1-Eyelid; C44.2-External ear; C44.3-Skin other/unspec parts of face; C44.4-Skin of scalp and neck; C44.5-Skin of trunk; C44.6-Skin of upper limb and shoulder; C44.7-Skin of lower limb and hip; C44.8-Overlapping lesion of skin; C44.9-Skin, NOS |
| Pulmonary | 1,500 | C33.9-Trachea; C34.0-Main bronchus; C34.1-Upper lobe, lung; C34.2-Middle lobe, lung; C34.3-Lower lobe, lung; C34.8-Overlapping lesion of lung; C34.9-Lung, NOS; C37.9-Thymus |
| Other Gastrointestinal | 1,487 | C15.3-Upper third of esophagus; C15.4-Middle third of esophagus; C15.5-Lower third of esophagus; C15.8-Overlapping lesion of esophagus; C15.9-Esophagus, NOS; C17.0-Duodenum; C17.1-Jejunum; C17.2-Ileum; C17.3-Meckels diverticulum; C17.8-Overlapping lesion of small intestine; C17.9-Small intestine, NOS; C18.0-Cecum; C18.1-Appendix; C18.2-Ascending colon; C18.3-Hepatic flexure of colon; C18.4-Transverse colon; C18.5-Splenic flexure of colon; C18.6-Descending colon; C18.7-Sigmoid colon; C18.8-Overlapping lesion of colon; C18.9-Colon, NOS; C19.9-Rectosigmoid junction; C20.9-Rectum, NOS; C21.0-Anus, NOS; C21.8-Overlapping lesion of rectum, anus, and anal canal; C22.0-Liver; C23.9-Gallbladder; C24.0-Extrahepatic bile duct; C24.1-Ampulla of Vater; C25.0-Head of pancreas; C25.2-Tail of pancreas; C25.9-Pancreas, NOS; C26.0-Intestinal tract, NOS; C26.8-Overlapping lesion of digestive system; C26.9-Gastrointestinal tract, NOS |
| Salivary Gland | 1,186 | C07.9-Parotid gland; C08.0-Submandibular gland; C08.1-Sublingual gland; C08.9-Major salivary gland, NOS |
| Oropharyngeal | 624 | C00.0-External upper lip; C00.1-External lower lip; C00.3-Mucosa of upper lip; C00.4-Mucosa of lower lip; C00.5-Mucosa of lip, NOS; C00.9-Lip, NOS; C01.9-Base of tongue, NOS; C02.1-Border of tongue; C02.3-Anterior 2/3 of tongue, NOS; C02.4-Lingual tonsil; C02.9-Tongue, NOS; C03.0-Upper gum; C03.1-Lower gum; C03.9-Gum, NOS; C04.1-Lateral floor of mouth; C04.9-Floor of mouth, NOS; C05.0-Hard palate; C05.1-Soft palate, NOS; C05.2-Uvula; C05.8-Overlapping lesion of palate; C05.9-Palate, NOS; C06.0-Cheek mucosa; C06.1-Vestibule of mouth; C06.2-Retromolar area; C06.9-Mouth, NOS; C09.0-Tonsillar fossa; C09.8-Overlapping lesion of tonsil; C09.9-Tonsil, NOS; C10.0-Vallecula; C10.1-Anterior surface of epiglottis; C10.3-Posterior wall of oropharynx; C10.8-Overlapping lesion of oropharynx; C10.9-Oropharynx, NOS; C11.0-Superior wall of nasopharynx; C11.1-Posterior wall of nasopharynx; C11.2-Lateral wall of nasopharynx; C11.3-Anterior wall of nasopharynx; C11.8-Overlapping lesion of nasopharynx; C11.9-Nasopharynx, NOS; C12.9-Pyriform sinus; C13.1-Aryepiglottic fold, hypopharyngeal; C13.9-Hypopharynx, NOS; C14.0-Pharynx, NOS; C14.2-Waldeyers ring; C14.8-Overlapping lesion of lip, oral cavity & pharynx; C30.0-Nasal cavity; C30.1-Middle ear; C31.0-Maxillary sinus; C31.1-Ethmoid sinus; C31.2-Frontal sinus; C31.8-Overlapping lesion of accessory sinuses; C31.9-Accessory sinus, NOS; C32.0-Glottis; C32.1-Supraglottis; C32.2-Subglottis; C32.9-Larynx, NOS |
| Breast | 576 | C50.0-Nipple; C50.1-Central portion of breast; C50.2-Upper-inner quadrant of breast; C50.3-Lower-inner quadrant of breast; C50.4-Upper-outer quadrant of breast; C50.5-Lower-outer quadrant of breast; C50.6-Axillary tail of breast; C50.8-Overlapping lesion of breast; C50.9-Breast, NOS |
| Bone/Connective Tissue | 383 | C40.0-Long bones: upper limb, scapula, and associated joints; C40.2-Long bones of lower limb and associated joints; C40.3-Short bones of lower limb and associated joints; C40.9-Bone of limb, NOS; C41.0-Bones of skull and face and associated joints; C41.1-Mandible; C41.2-Vertebral column; C41.3-Rib, sternum, clavicle and associated joints; C41.4-Pelvic bones, sacrum, coccyx and associated joints; C41.9-Bone, NOS; C47.1-Periph nerves & autonomic nerv sys: upr limb, shoulder; C47.9-Autonomic nervous system, NOS; C49.0-Conn, subcutaneous, other soft tis: head, face, neck; C49.1-Conn, subcutaneous, other soft tis: upr limb, shoulder; C49.2-Conn, subcutaneous, other soft tis: lower limb, hip; C49.3-Conn, subcutaneous, other soft tis: thorax; C49.4-Conn, subcutaneous, other soft tis: abdomen; C49.5-Conn, subcutaneous, other soft tis: pelvis; C49.6-Conn, subcutaneous, other soft tis: trunk, NOS; C49.8-Overlap conn, subcutaneous, and other soft tissues; C49.9-Conn, subcutaneous and other soft tissues, NOS |
| Thyroid | 294 | C73.9-Thyroid gland |
| Genitourinary | 265 | C51.9-Vulva, NOS; C52.9-Vagina, NOS; C53.0-Endocervix; C53.9-Cervix uteri; C54.1-Endometrium; C54.9-Corpus uteri; C56.9-Ovary; C57.4-Uterine adnexa; C57.8-Overlapping lesion of female genital organs; C61.9-Prostate gland; C62.9-Testis, NOS; C63.0-Epididymis; C63.2-Scrotum, NOS; C64.9-Kidney, NOS; C65.9-Renal pelvis; C66.9-Ureter; C67.0-Trigone of bladder; C67.1-Dome of bladder; C67.2-Lateral wall of bladder; C67.3-Anterior wall of bladder; C67.4-Posterior wall of bladder; C67.5-Bladder neck; C67.8-Overlapping lesion of bladder; C67.9-Bladder, NOS; C68.0-Urethra; C68.8-Overlapping lesion of urinary organs; C68.9-Urinary system, NOS |
| Other | 141 | C38.0-Heart; C38.1-Anterior mediastinum; C38.2-Posterior mediastinum; C38.3-Mediastinum, NOS; C38.4-Pleura, NOS; C48.0-Retroperitoneum; C48.1-Specified parts of peritoneum; C48.2-Peritoneum, NOS; C74.9-Adrenal gland, NOS; C75.0-Parathyroid gland; C75.8-Overlapping lesion of endocrine glands; C76.0-Head, face or neck, NOS; C76.1-Thorax, NOS; C76.2-Abdomen, NOS; C76.3-Pelvis, NOS; C76.4-Upper limb, NOS; C76.5-Lower limb, NOS |
| Unknown | 119 | C80.9-Unknown primary site |
